# Holistic General Practice: A Student`s Perspective

**DOI:** 10.1080/17571472.2015.1113714

**Published:** 2015-12-24

**Authors:** Olivia Martin

**Affiliations:** ^a^ Barts and the London School of Medicine & Dentistry, Queen Mary University of London, Turner St, Greater London, UK

**Keywords:** General practice, medical student, holistic medicine

## Abstract

This patient was a lady in her 50s who had previously been diagnosed with anxiety, depression and rectal cancer. She had undergone neo-adjuvant chemotherapy designed to shrink the tumour before it was removed surgically. However, the cancer responded better than expected to the chemotherapy, and the oncologists could no longer find evidence of active cancer within the patient. The oncologist had recently been speaking with the patient about whether or not she should continue with the surgery they had originally planned. The oncologist was keen to go ahead with the surgery, as it offered the best chance of long term disease free survival. The patient, however, was extremely nervous about going through major surgery, and was less sure that it was the right option now that she had been told that there was no evidence of active cancer. However, she had been unable to come to a definitive decision despite 3 consultations with her oncologist and the surgeons ready to perform the operation and the oncologist had written to the patients GP to this effect. This patient taught me a variety of things. Firstly, the background given to me about this patients’ first consultation opened my eyes to the importance of certain parts of the medical history. For example, the rectal cancer was only picked up due to additional symptoms found during the review of systems. The patient consultation that I witnessed was regarding a decision the patient was struggling to make over whether or not to have surgery following seemingly successful neo-adjuvant chemotherapy. The patient was struggling to make this decision due to a lack of support and the GP had scheduled this appointment to help her come to terms with the decision that she needed to make.

## Why this matters to me

Observing this consultation really opened my eyes to the work that General Practitioners perform on a daily basis. Not only are they the first point of contact for almost every patient seen in the NHS, they are also co-ordinators of care and are more responsible for each patients’ long term, overall health and well-being than most hospital doctors. Prior to this consultation, I think I was quite naïve about how varied the role of a GP is. General Practice is an area that I have always considered high on my list of potential career pathways, but as a medical student you don’t seem to get a very clear idea of what GPs actually do. I think this is partly due to the fact that our placements are usually based around a clinical module, for example cardiorespiratory, so we tend to see patients related to that topic.

## Key messages

• General Practice is more than an entry way for patients to Secondary Care. The role of a General Practitioner is complex and varied, and will be different for different patients.
• Patients require more than medical treatment, look at the patient as a whole person rather than a set of symptoms. A holistic approach is needed to provide adequate treatment.
• Medical students often do not get a very clear picture of what the job of a General Practitioner is. They are often left out of more difficult consultations, or only shown those relevant to the system that they are currently studying.

Whilst at a GP surgery in East London, I witnessed a consultation that completely challenged my understanding of the role of General Practitioners.

The patient was a lady in her 50s who had previously been diagnosed with anxiety, depression and rectal cancer. She had first come to the surgery with difficulty sleeping, but altered bowel habit had been picked up during a review of systems and the diagnosis was subsequently made. She had recently undergone chemotherapy designed to shrink the tumour before it was removed surgically. However, the cancer responded far better than expected to the chemotherapy, and there was no evidence of active cancer within the patient. The oncologist had recently been speaking with the patient about whether or not she should continue with the surgery they had originally planned. The oncology team was keen to go ahead with the surgery, as it offered the best chance of long-term disease-free survival. The patient, however, was extremely nervous about going through major surgery, and was less certain that it was the right option now that she had been told that there was no evidence of active cancer. Despite several hospital consultations with her oncologist and the surgeons ready to perform the operation, the patient was unable to come to a definitive conclusion about the surgery, and continued to put off making the decision. Eventually, the oncologist had written to the patients GP explaining that she had not yet decided what to do.

The appointment that I witnessed had been scheduled by the GP in response to the letter from the oncologist.

The GP started by asking the patient how she was feeling, and eased into the consultation with some general questions about her exercise ability and diet. This allowed the patient to feel comfortable despite my presence.

The GP asked the patient how she was feeling about her recent treatment. The patient explained her relief that there was no evidence of cancer in her rectum or elsewhere in her body, and her anxiety with regards to the surgery that her oncologist was still proposing. She explained that she was trying not to think about the surgery because she couldn’t make a decision, and felt confused about the best course of action. As the GP asked a few more questions about side effects and complications she might have experienced, the patient relaxed again, so the questions circled back around to the surgery. The patient quite openly discussed how she was weighing the pros and cons of having surgery for a cancer that may have been cured against the possibility of it returning.

This exchange between the GP and patient was extremely interesting to me. I had previously always been wary of discussing future treatments with patients, as I was always afraid that I might be asked for my own opinion on their condition or options. I don’t feel qualified to give a valid opinion so early in my training. I also worry that my own opinion would affect their decision, because even as medical students we represent the medical profession, and our opinions can be taken differently in the context of a hospital than over a cup of tea with a friend.

In this conversation however the patient never once asked for the GPs opinion or advice, despite the fact that they clearly had a good relationship, with a great deal of respect on both sides. I believe that the reason that it never came up is because of the way that the GP led the conversation, asking how the patient thought, and what she felt and which way she was leaning. This meant that the patient explored her own feelings rather than relying on anyone else’s ideas and opinions. The GP also encouraged the patient to talk about her future, both with and without the surgery. This led to the patient to discuss who she could call on to help her if she went through with the surgery, or if she chose not to and became ill again.

After the patient left, the GP asked me a question: ‘Why do you think I scheduled that appointment?’

I wasn’t really sure at first and replied ‘To see how the patient was getting on after her treatment?’

The GP laughed and said that she had access to the patients’ hospital records, and could easily have checked on her by phone rather than asking her to travel to the surgery.

After thinking about it, I realised that the main thing that the patient had got out of the consultation was to start really thinking about the decision that she needed to make. The GP explained that the patient did not get on with her family, had given up work due to her depression and as a result was very isolated. This meant that she felt she did not have anyone close to her that she could discuss her options with, without fear of an argument or confrontation causing additional stress. When the patients’ oncologist had written to the GP regarding the patients’ reluctance to make a decision over whether or not to continue with the surgery, the GP had decided to call the patient in simply to ensure that she was really thinking about whether or not the surgery was the right thing for her.

This consultation was an excellent one for me to see at this stage of my career. It was the first consultation that I had witnessed where the patient hadn’t come in looking for a diagnosis or medication. Essentially the patient hadn’t come in looking for anything. However, she received a lot of help and support and left the consultation having really thought about the decision that she needed to make. On top of this, by asking about people she could call on to help after the surgery, the GP had made the patient aware of the support systems that she did have in place, which were clearly not obvious to her prior to this conversation.

This is a vital part of the job of the General Practitioner, and one that cannot be taught in lectures. It is easy to see GP work as an algorithm of history, investigations, diagnosis and management but actually the role is about treating the whole patient. Where a cardiologist checks that the heart is pumping, and a gastroenterologist keeps the bowels moving, a general practitioner has to take the whole patient as they come. This is what makes the role a very different challenge to hospital medicine. This is also evidenced by the fact that the patients rectal cancer diagnosis came about after an appointment about troubled sleep!

The main thing that I learned from this consultation was that when trying to treat a patient it is important to remember that two patients in an identical situation will handle it completely differently. These differences are due to a combination of their support systems, their home circumstances, prior experiences and their personality. For this lady, who felt she had no one to talk to, she struggled to make a decision and so she began to ignore the problem and continue to see her surgeon and oncologist without coming to a conclusion. Her anxiety also prevented her from being confident in any decision she made, so she changed her mind repeatedly. Others may find this decision easier to make based on their expectations for their lives, the responsibilities they have and the help that they may receive from those around them. This is an important insight that I couldn’t really have learned and understood at medical school, and one that I hope will shape my interaction with my patients throughout my career.

